# Systemic Sclerosis Presenting With Long-Standing Peripheral Neuropathy and Delayed Multisystem Manifestations: A Case Report

**DOI:** 10.7759/cureus.116119

**Published:** 2026-09-11

**Authors:** Bhargavan PV, Sila Chandran, Aswin US, Shine Antony, Meera Bhagyanathan

**Affiliations:** 1 Department of Internal Medicine, Baby Memorial Hospital, Calicut, IND; 2 Department of Dermatology, Baby Memorial Hospital, Calicut, IND

**Keywords:** charcot arthropathy, gastric antral vascular ectasia, interstitial lung disease, overlap connective tissue disease, peripheral neuropathy, pulmonary arterial hypertension, systemic sclerosis

## Abstract

Systemic sclerosis (SSc) is a rare autoimmune connective tissue disorder characterized by immune dysregulation, microvascular injury, and progressive fibrosis involving the skin and internal organs. Although Raynaud phenomenon and cutaneous sclerosis are the hallmark manifestations, atypical presentations may significantly delay diagnosis. Peripheral neuropathy is an uncommon neurological manifestation and rarely precedes the diagnosis of SSc by several years.

We report the case of a 62-year-old female who presented with a 20-year history of progressive distal paresthesia involving both upper and lower limbs, followed by recurrent lower limb ulceration and progressive unilateral lower-limb swelling associated with skin thickening and deformity. Nearly two decades after the onset of neurological symptoms, she developed progressive dysphagia, constitutional symptoms, exertional dyspnea, and pigmentary skin changes. Evaluation demonstrated positive antinuclear antibodies with strong anti-Ro52 and anti-RNP/Sm antibodies, mixed sensorimotor peripheral neuropathy with bilateral carpal tunnel syndrome, pulmonary arterial hypertension, interstitial lung disease, moderate pericardial effusion, gastric antral vascular ectasia, and histopathological evidence of dermal sclerosis. She was diagnosed with SSc with overlap autoimmune features and managed through a multidisciplinary approach involving rheumatology, pulmonology, gastroenterology, rehabilitation medicine, and physiotherapy.

This case illustrates an unusual neuropathy-predominant presentation in which peripheral nerve involvement preceded classical systemic manifestations by almost two decades. It highlights the importance of considering connective tissue diseases in patients presenting with chronic unexplained peripheral neuropathy, recurrent trophic ulceration, or progressive localized skin sclerosis. Early recognition of such atypical presentations may facilitate timely diagnosis, comprehensive organ screening, and administration of disease-modifying therapy before irreversible organ damage develops.

## Introduction

Systemic sclerosis (SSc) is a chronic multisystem autoimmune connective tissue disease characterized by immune dysregulation, endothelial dysfunction, progressive microvascular injury, and excessive collagen deposition leading to fibrosis of the skin and multiple internal organs. The disease exhibits marked clinical heterogeneity, ranging from limited cutaneous involvement to diffuse systemic disease with severe pulmonary, gastrointestinal, cardiac, and renal complications. Pulmonary arterial hypertension (PAH) and interstitial lung disease (ILD) remain the leading causes of disease-related mortality, underscoring the importance of early diagnosis and systematic screening for visceral involvement [1-3].

The classical clinical evolution often begins with Raynaud’s phenomenon, followed by puffy fingers and progressive skin thickening, which may subsequently result in sclerodactyly, contractures, digital ischemia and ulcers, telangiectasia, calcinosis, and pigmentary abnormalities. However, atypical manifestations involving the musculoskeletal or nervous system may precede classical features by several years, frequently resulting in delayed diagnosis [3,4].

SSc is broadly categorized as limited cutaneous SSc (lcSSc) or diffuse cutaneous SSc (dcSSc) based on the extent of skin involvement. In lcSSc, skin thickening is generally restricted to areas distal to the elbows and knees, with involvement of the face and sparing of the trunk; patients may have a prolonged period of Raynaud’s phenomenon before the development of other manifestations. In dcSSc, skin thickening extends proximal to the elbows and/or knees and may involve the trunk. Diffuse disease generally follows a more rapidly progressive course and is more strongly associated with early clinically significant internal-organ involvement, particularly ILD, gastrointestinal disease, cardiac involvement, and renal complications. Nevertheless, these phenotypes represent broad clinical patterns rather than rigid disease trajectories, and the timing, severity, and combination of organ manifestations vary considerably between individual patients [3,4].

Peripheral nervous system involvement has traditionally been considered uncommon in SSc, although studies suggest that both large-fiber and small-fiber neuropathies are likely underrecognized. Proposed mechanisms include ischemic injury secondary to obliterative microangiopathy of the vasa nervorum, immune-mediated nerve injury, tissue fibrosis causing compressive neuropathies, and associated overlap autoimmune syndromes [2,4-6].

Charcot neuroarthropathy is an uncommon manifestation in SSc and is generally associated with longstanding peripheral neuropathy. Likewise, gastric antral vascular ectasia (GAVE), severe pulmonary arterial hypertension, and clinically significant pericardial effusion represent uncommon but important markers of advanced systemic disease [7-9].

We report a rare case of SSc with approximately two decades of progressive peripheral neuropathy and recurrent trophic ulceration, in whom the neurological manifestations preceded the diagnosis of diabetes mellitus by approximately ten years. Over the subsequent years, she developed progressive skin sclerosis and pigmentary abnormalities, followed by gastrointestinal, pulmonary, and cardiac manifestations. The prolonged neurological presentation and atypical temporal sequence initially broadened the differential diagnosis, including diabetic neuropathy and leprosy. This case highlights the importance of recognizing peripheral neuropathy as a potentially multifactorial manifestation occurring within the broader clinical spectrum of SSc and emphasizes the need for periodic diagnostic reassessment when chronic neurological symptoms coexist with evolving connective tissue disease manifestations.

## Case presentation

A 62-year-old homemaker presented with a progressively evolving multisystem illness spanning nearly two decades. Her symptoms began approximately 20 years before presentation with intermittent tingling and numbness involving both palms and soles. Initially mild and episodic, these sensory symptoms gradually progressed in severity and became persistent, interfering with routine daily activities. Three years after the onset of neuropathic symptoms, she developed recurrent painless ulcerations over the left foot that healed slowly and recurred despite conservative treatment.

During the same period, she noticed progressive swelling of the left foot and leg below the knee. The swelling was initially intermittent but gradually became persistent and was associated with progressive skin thickening, hyperpigmentation, induration, and deformity of the foot. Over the following years, similar but less severe swelling developed in the contralateral lower limb. The left foot progressively shortened, with deformity of the toes and subluxation of the metatarsophalangeal joints, eventually resulting in difficulty walking associated with recurrent ulcers and destruction of the nails (Figure 1). 

**Figure 1:**
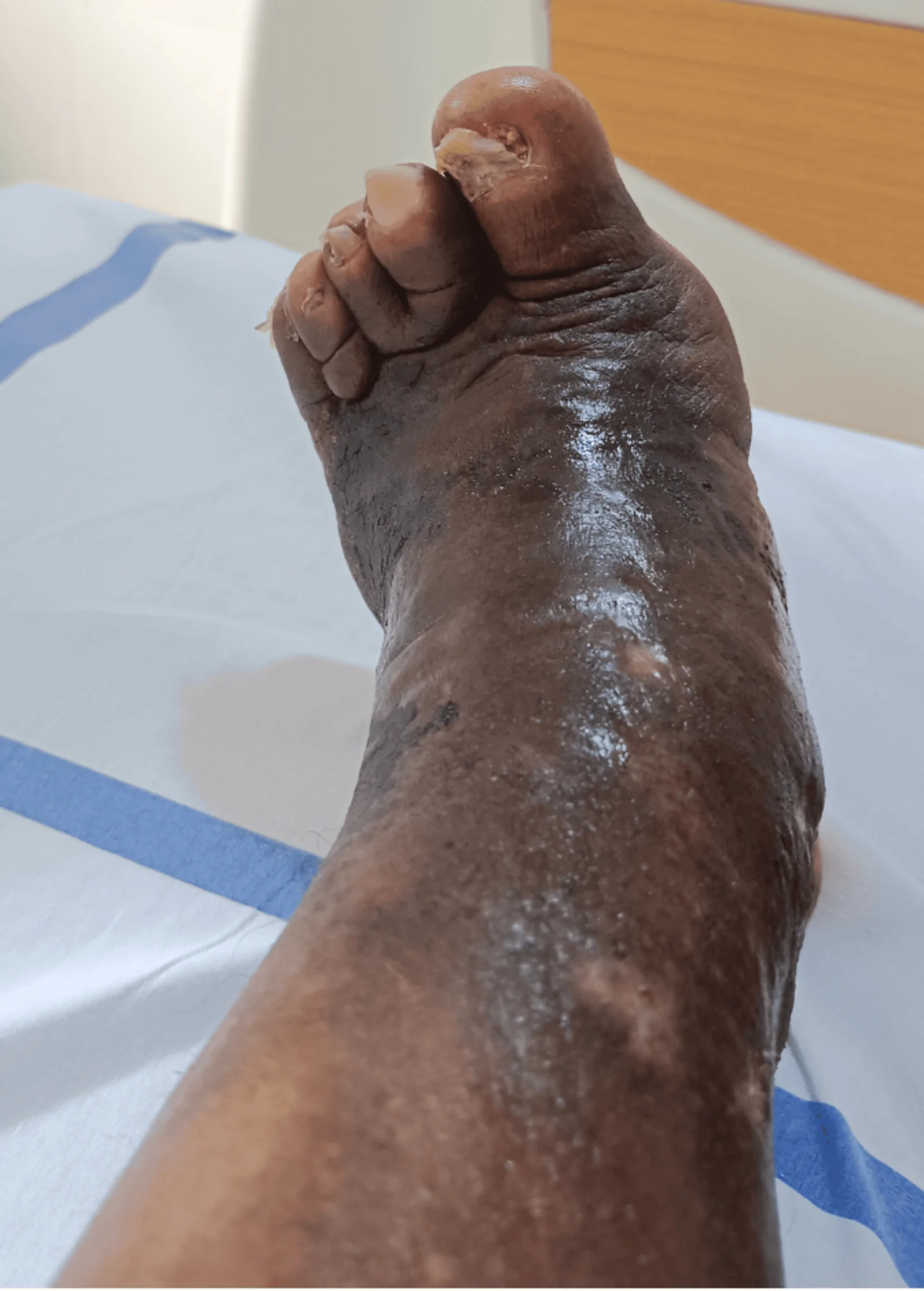
Left foot Left foot showing significant non-pitting edema, deformed toes, destroyed nails, and multiple ulcers.

Approximately one year before admission, she developed gradually progressive dysphagia involving both solids and liquids. The dysphagia was associated with reduced appetite and an unintentional weight loss of nearly 10 kg over one year. Two months before presentation, she began experiencing progressive exertional dyspnea corresponding to New York Heart Association functional class II-III, without orthopnea or paroxysmal nocturnal dyspnea. She also noticed multiple hypopigmented macules over the scalp, forehead, retroauricular region (Figure 2), forearms, hands, and lower limbs, giving a characteristic salt-and-pepper appearance with sparing of the trunk. There was no history of Raynaud phenomenon, photosensitivity, oral ulcers, proximal muscle weakness, inflammatory arthritis, recurrent miscarriages, or thromboembolic events.

**Figure 2:**
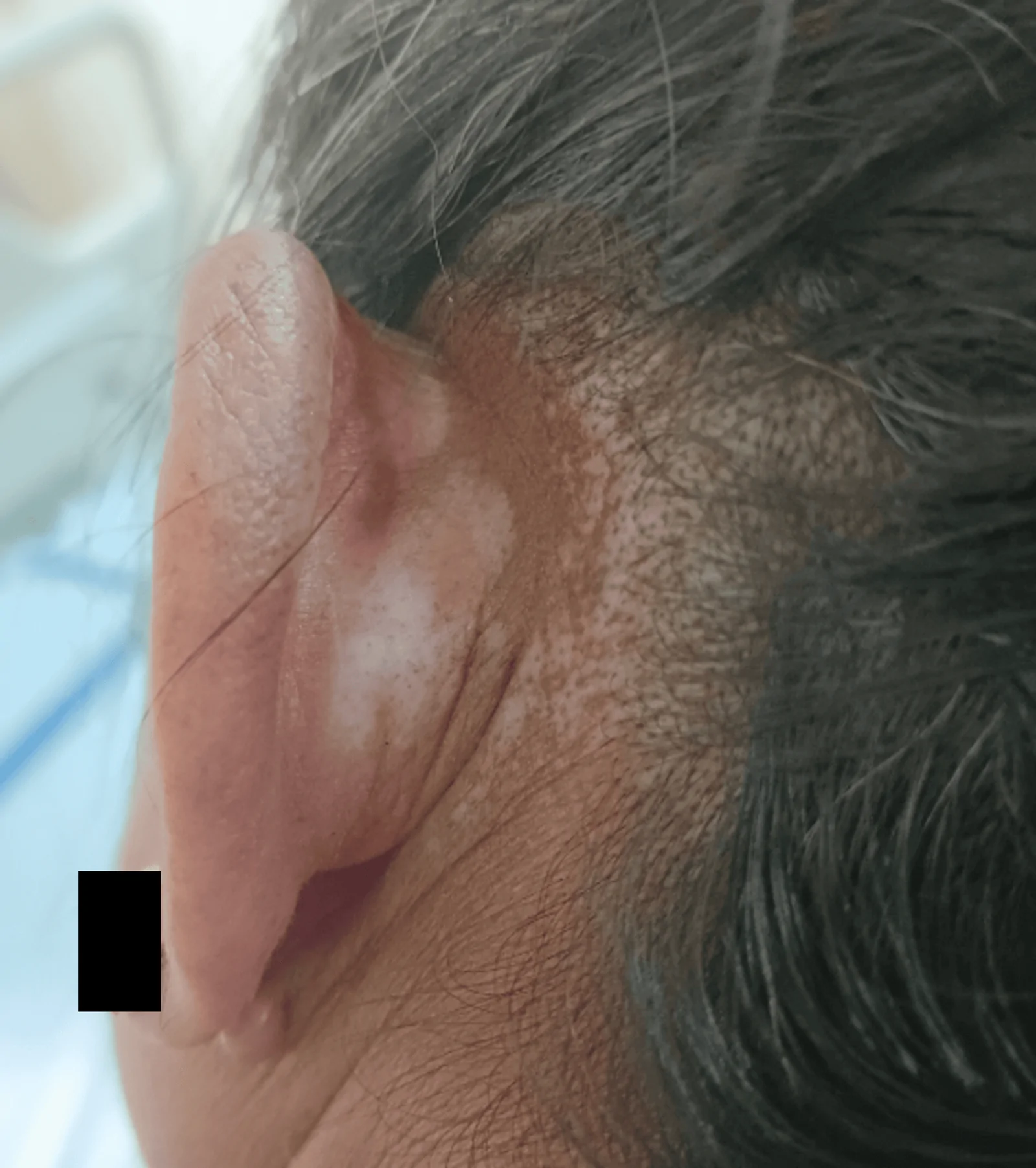
Salt-and-pepper pigmentation involving retroauricular region and scalp

The patient’s medical history was significant for well-controlled type 2 diabetes mellitus, systemic hypertension, and primary hypothyroidism, all managed with regular medications. She denied any history of tuberculosis, leprosy, inflammatory bowel disease, chronic alcohol consumption, smoking, exposure to neurotoxins, or a family history of autoimmune or connective tissue diseases.

On examination, she appeared chronically ill and malnourished and had pallor and bilateral nonpitting pedal edema (left > right). Dermatological examination revealed characteristic salt-and-pepper pigmentation involving the face, retroauricular region (Figure 2), and lower limbs, interspersed with multiple hypopigmented macules. Diffuse induration and hyperpigmentation of the skin were confined predominantly to the left lower limb below the knee, producing marked asymmetry between the two lower extremities. The affected skin was thickened, shiny, and tightly adherent to the underlying tissues (Figure 3). Multiple healed trophic scars and one active plantar ulcer were noted over the right foot (Figure 4). Puffy fingers were present bilaterally without obvious digital gangrene or calcinosis cutis (Figure 5).

**Figure 3:**
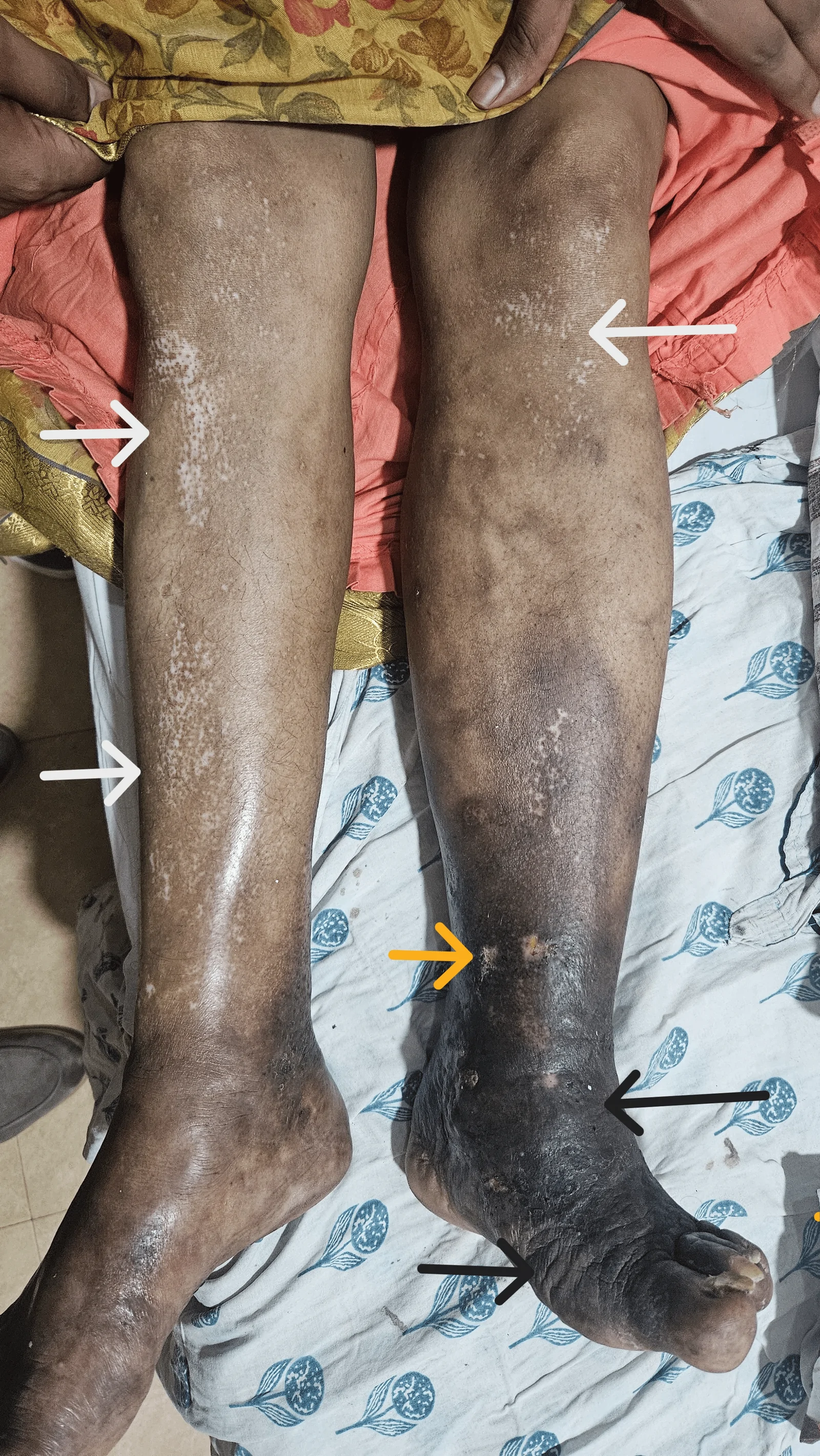
Bilateral lower limb The figure shows asymmetry between the two limbs, salt-and-pepper pigmentation (white arrows), induration, skin thickening (black arrow), and a healed trophic ulcer (yellow arrow).

**Figure 4:**
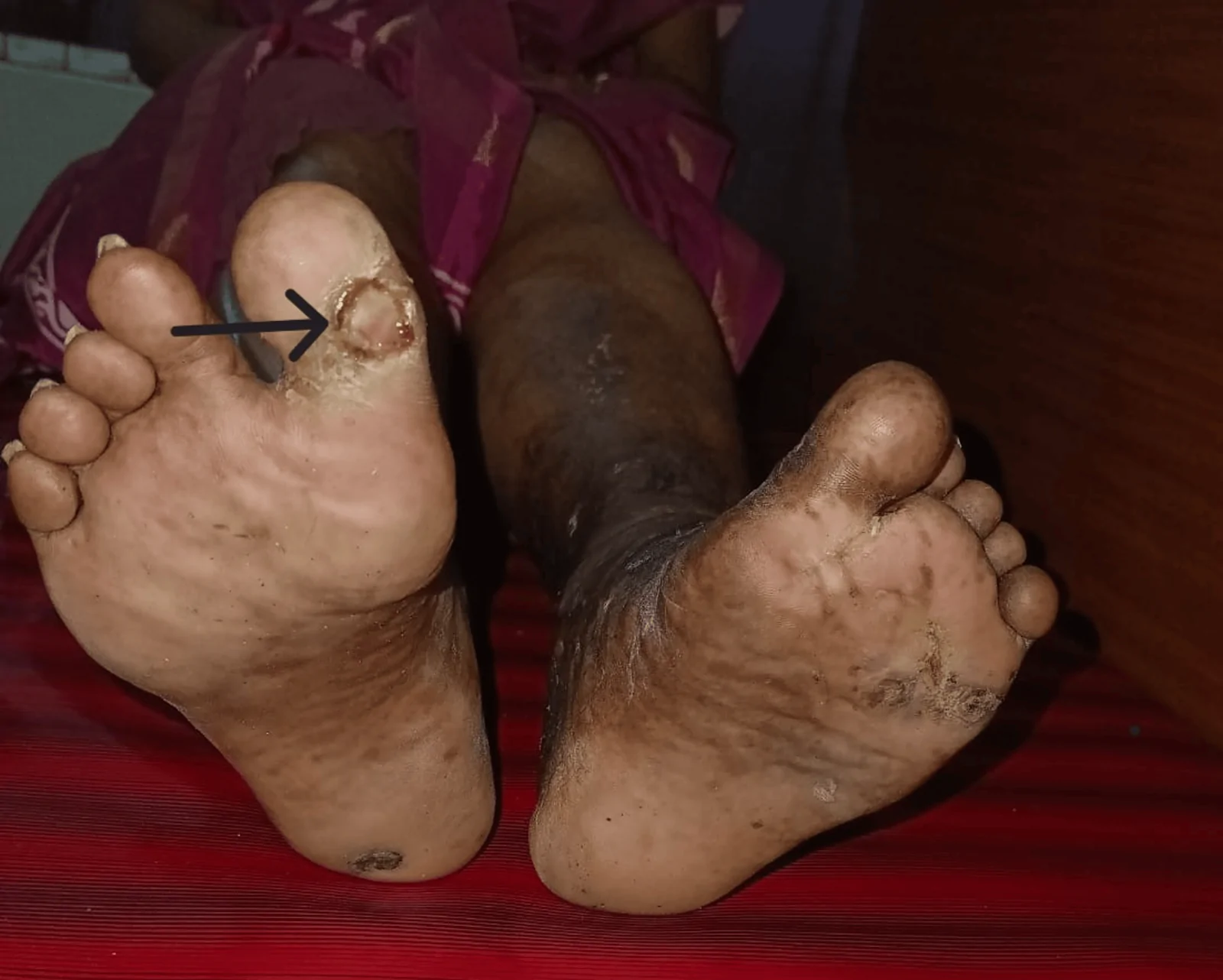
Plantar aspect of bilateral lower limb The figure shows an active ulcer on the right foot (black arrow).

**Figure 5:**
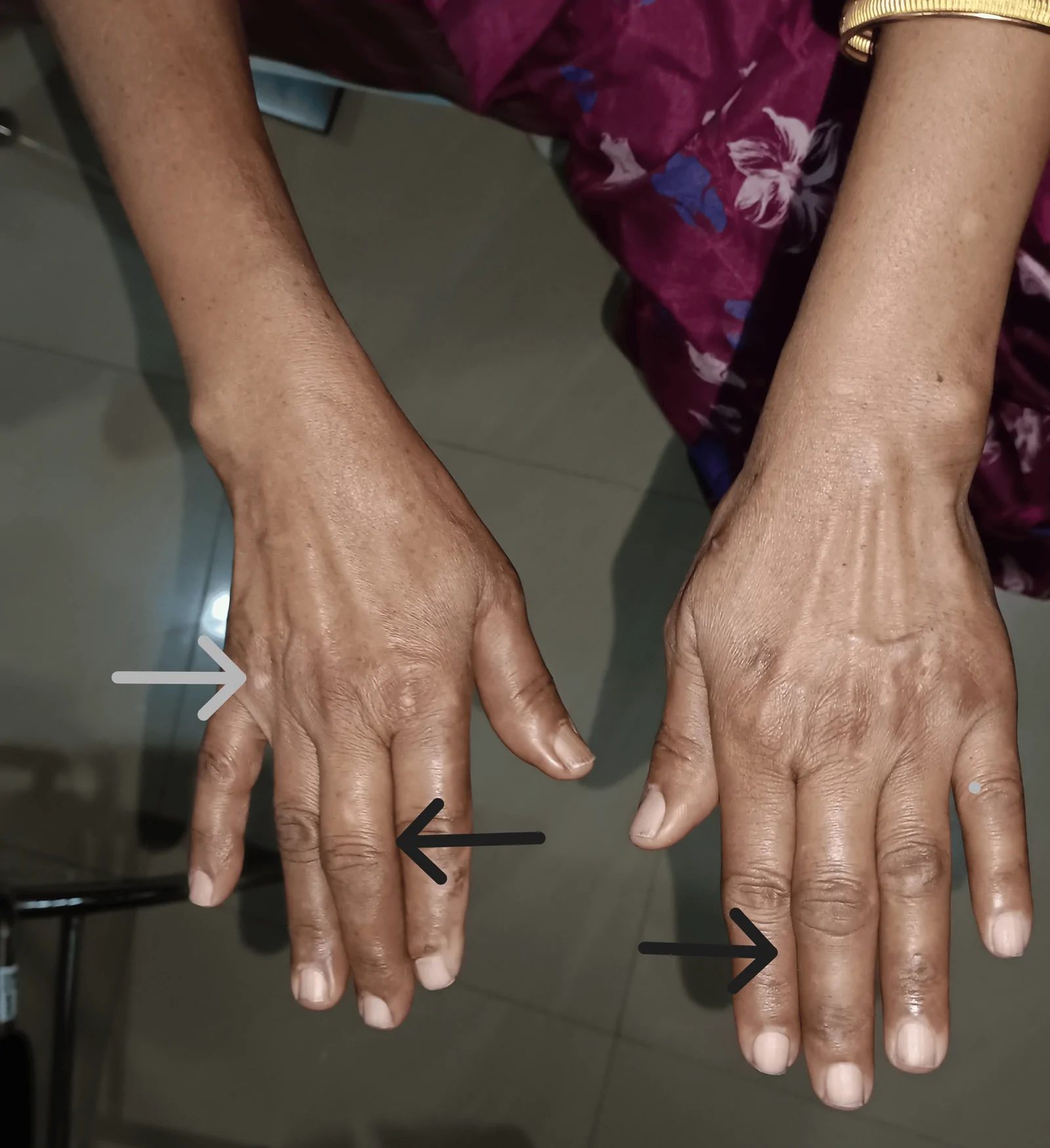
Bilateral hands The figure shows puffy fingers, left > right (black arrows), and a hypopigmented macule on the right hand (white arrow).

Examination of the musculoskeletal system demonstrated deformity of the left foot with shortening of the forefoot (Figure 3), clawing of the toes, and subluxation of the metatarsophalangeal joints consistent with chronic neuropathic arthropathy. Range of motion of the ankle and metatarsophalangeal joints was restricted because of fibrosis and deformity.

Neurological examination revealed symmetrical stocking-distribution impairment of pain, temperature, vibration, and joint position sensation in the bilateral lower limbs. Motor examination revealed mild distal weakness of the intrinsic foot muscles with preserved proximal muscle strength. Deep tendon reflexes were absent at the ankles, while knee jerks were preserved. Plantar responses were flexor bilaterally. Romberg’s sign was positive, indicating significant proprioceptive impairment. Cranial nerve examination was normal, and there were no cerebellar signs.

Cardiovascular examination demonstrated a prominent pulmonary component of the second heart sound without audible murmurs. Respiratory examination revealed fine bilateral end-inspiratory crackles over the mammary, infra-axillary, and infrascapular areas, while abdominal examination was unremarkable.

Investigations

Initial laboratory investigations demonstrated normocytic normochromic anemia with rouleaux formation on peripheral smear and an elevated erythrocyte sedimentation rate. Serum creatine phosphokinase was mildly elevated, suggesting possible subclinical inflammatory muscle involvement. Urinalysis demonstrated mild proteinuria with an elevated urine protein-creatinine ratio, although serum creatinine and estimated glomerular filtration rate remained within normal limits.

Autoimmune evaluation revealed positive antinuclear antibodies with strong positivity for anti-Ro52 and anti-RNP/Sm antibodies. Rheumatoid factor was also positive. Although anti-topoisomerase I (Scl-70) and anticentromere antibodies are the classical serological markers of SSc, their absence did not exclude the diagnosis because overlap syndromes frequently demonstrate alternative autoantibody profiles.

**Table 1:** Laboratory investigations

Investigation	Result	Reference Range
Hemoglobin (Hb)	11.2	12-16 g/dL
Erythrocyte sedimentation rate (ESR)	52	<20 mm/hr
C-reactive protein (CRP)	4.1	0-5 mg/L
Vitamin B12	484	206-678
Serum iron	20	50-170 µg/dL
Total iron-binding capacity (TIBC)	273	250-450 µg/dL
Ferritin	148.9	10-120
HbA1c	5.6	<5.7%
Peripheral smear	Normocytic normochromic anemia with rouleaux formation	—
Serum creatinine	0.9	<1.4 mg/dL
Urine protein-creatinine ratio	1.29	<0.2 mg/mg
Thyroid-stimulating hormone (TSH)	4.881	0.4-4.5
FreeT4 (fT4)	1.01	0.56-1.5
Serum creatine phosphokinase (CPK)	190	34-145 U/L
Anti-Ro52	Positive (+++)	—
Anti-RNP/Sm	Positive (+++)	—
Rheumatoid factor (RF)	60.4	<14 IU/mL
Anti-topoisomerase I (Scl-70)	Negative	—
Anticentromere antibody (ACA)	Negative	—

Nerve conduction studies demonstrated mixed sensorimotor peripheral neuropathy involving both lower limbs together with bilateral median nerve entrapment at the wrists, consistent with carpal tunnel syndrome. These findings correlated well with the patient's long-standing sensory symptoms and trophic ulceration.

Plain radiography of the left foot demonstrated extensive destruction of the metatarsal joints and phalanges, with subluxation of multiple metatarsophalangeal and interphalangeal joints, consistent with Charcot neuroarthropathy (Figure 6).

**Figure 6:**
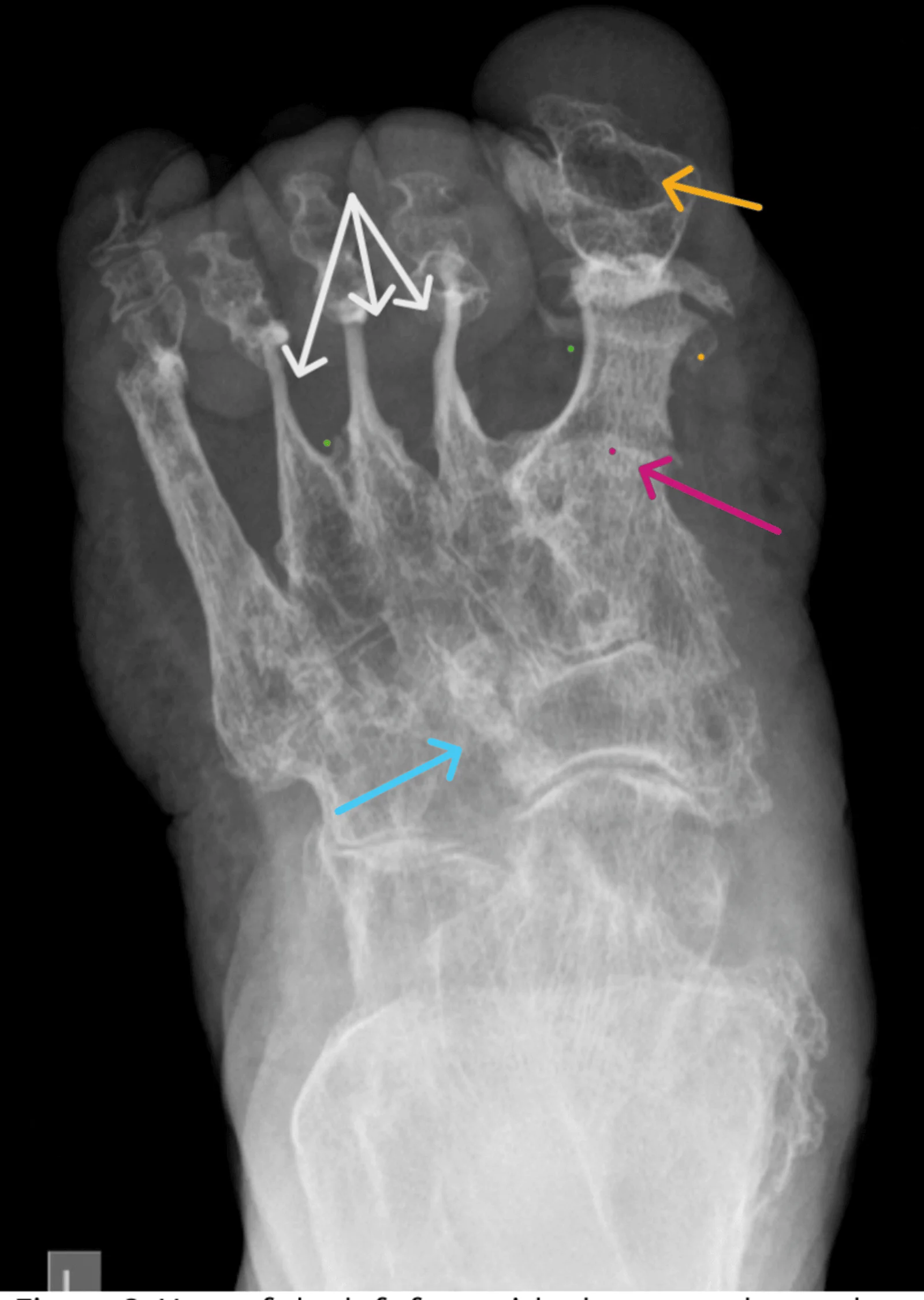
X-ray of left foot (AP) with Charcot arthropathy The figure shows advanced destruction of tarsals, metatarsals, and phalanges (yellow arrow). Licked-candy-stick appearance of the distal shaft, neck, and head of the 2nd, 3rd, and 4th metatarsals (white arrow). Extensive joint space narrowing at the tarsometatarsal (blue arrow) and metatarsophalangeal joints (pink arrow).

Histopathological examination of a skin biopsy obtained from the left hand demonstrated dense hyalinized collagen bundles replacing the normal dermis, with loss of adnexal structures, findings characteristic of scleroderma (Figure 7).

**Figure 7:**
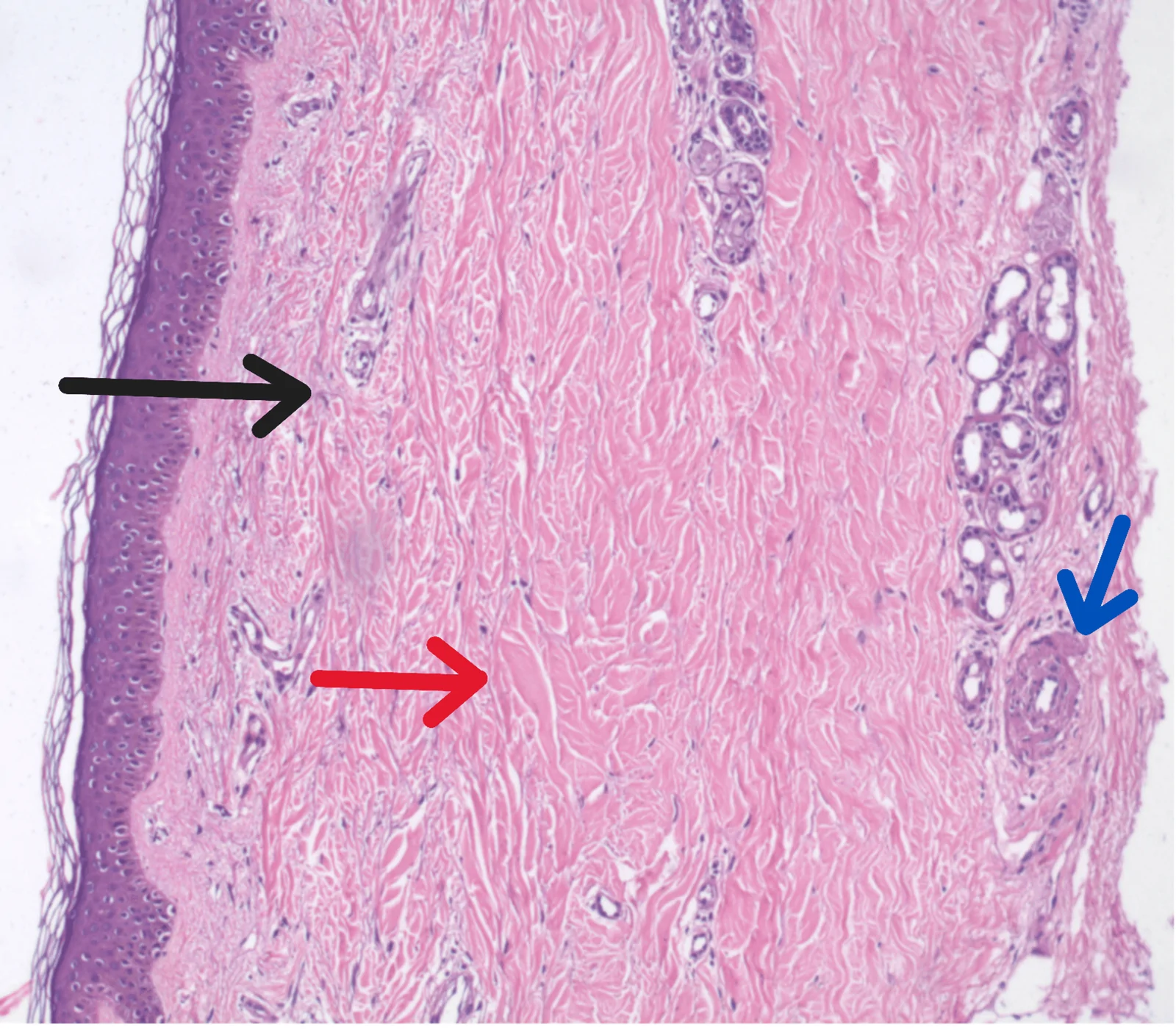
Histopathological section from the left hand with hematoxylin and eosin staining at 10× magnification The figure shows atrophic dermis (black arrow), hyalinised collagen in the dermis (red arrow), and pushed-up appendages (blue arrow) suggestive of scleroderma.

High-resolution computed tomography (HRCT) of the thorax demonstrated bilateral ground-glass opacities, interlobular septal thickening, traction bronchiectatic changes, and dilatation of the main pulmonary arteries, findings consistent with early ILD (Figure 8) complicated by pulmonary arterial hypertension and pericardial effusion (Figures 9, 10).

**Figure 8:**
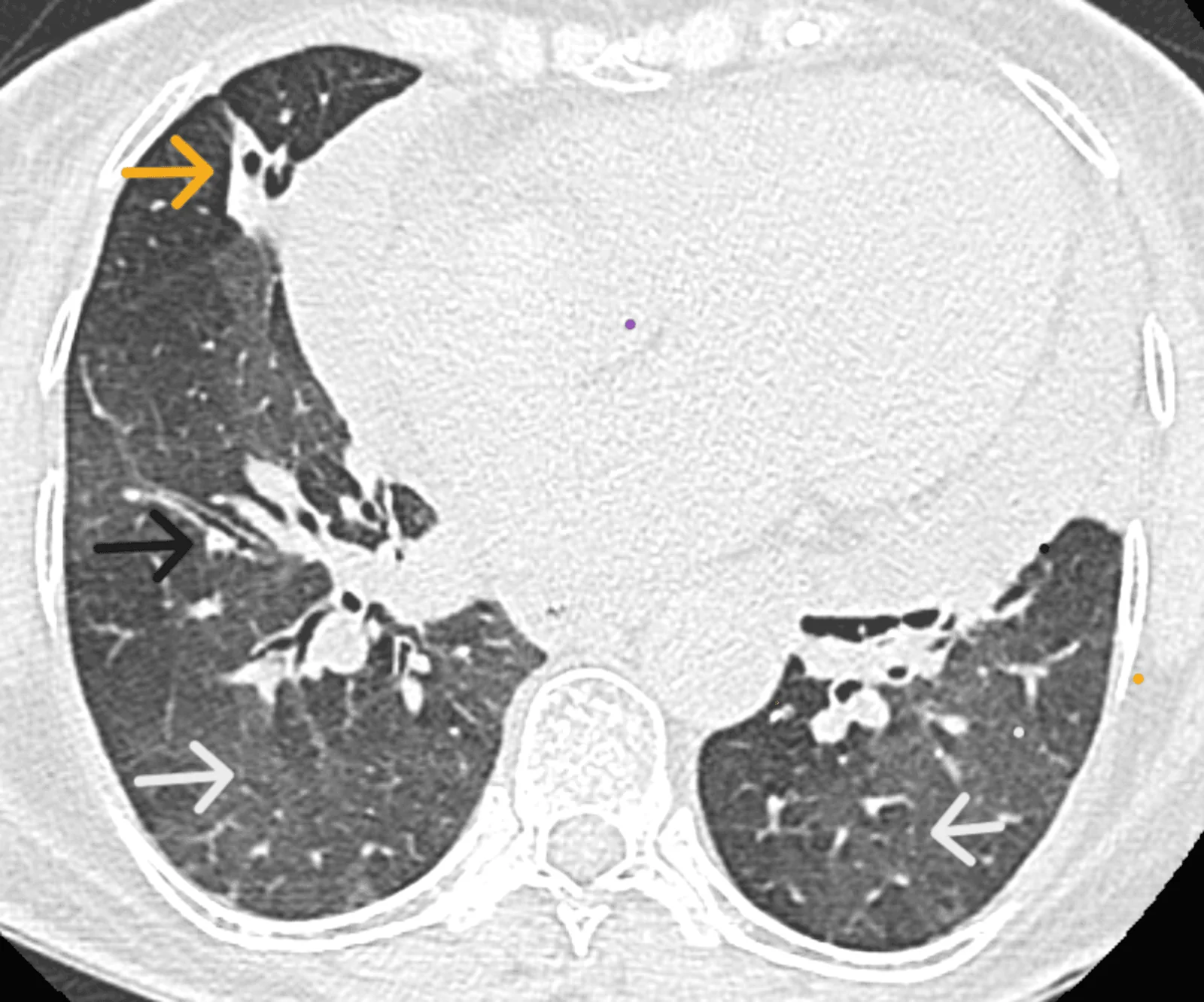
HRCT thorax (lung window) The figure shows bilateral ground-glass opacities (white arrow), interlobular septal thickening (yellow arrow), and traction bronchiectatic changes (black arrow) consistent with early interstitial lung disease.

**Figure 9:**
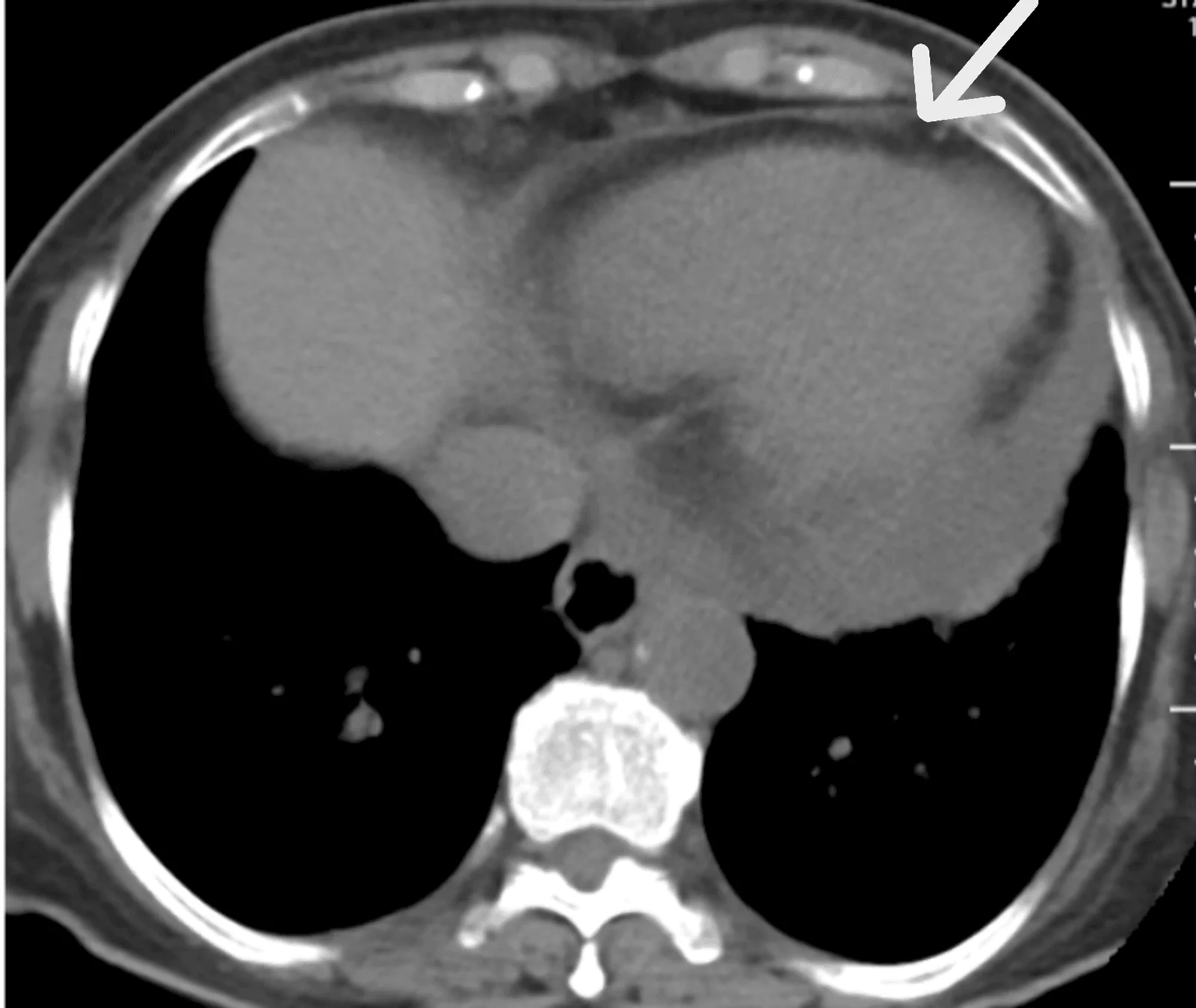
HRCT thorax (mediastinal window) CT film shows pericardial effusion (white arrow). HRCT: high-resolution computed tomography.

**Figure 10:**
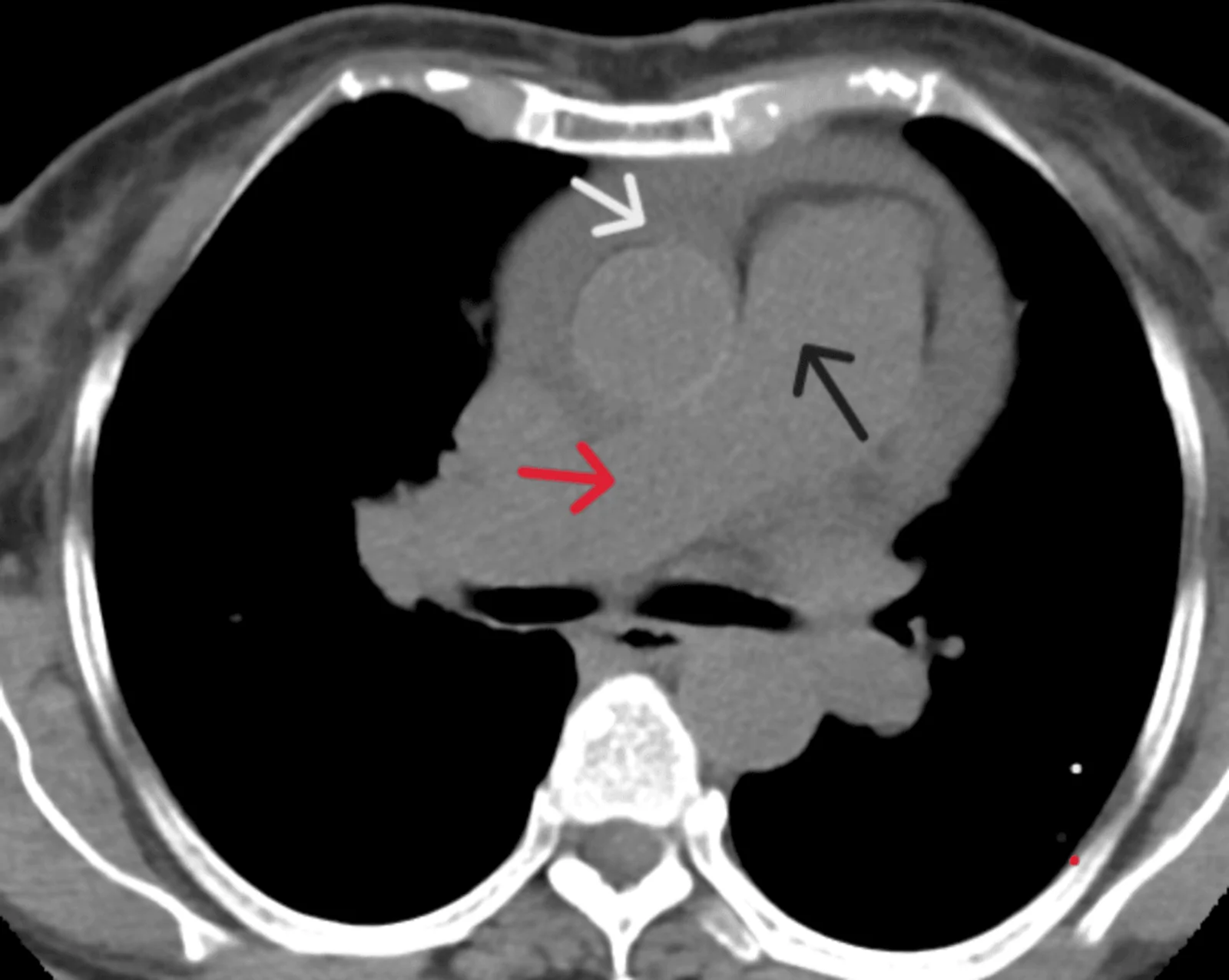
Axial section plain CT thorax (mediastinal window) The figure shows a dilated pulmonary trunk (black arrow) and right main pulmonary artery (red arrow). There is a mild increase in fluid within the pericardial recesses (white arrow).

Transthoracic echocardiography demonstrated moderate pulmonary arterial hypertension together with moderate circumferential pericardial effusion without evidence of cardiac tamponade. Left ventricular systolic function was preserved.

Pulmonary function testing demonstrated a mixed obstructive and restrictive ventilatory defect, correlating with the radiological evidence of ILD.

Upper gastrointestinal (GI) endoscopy performed to evaluate progressive dysphagia and anemia demonstrated gastric antral vascular ectasia (GAVE) (Figure 11), also known as “watermelon stomach,” a well-recognized vascular manifestation of SSc. The esophageal symptoms were considered secondary to scleroderma-related esophageal dysmotility.

**Figure 11:**
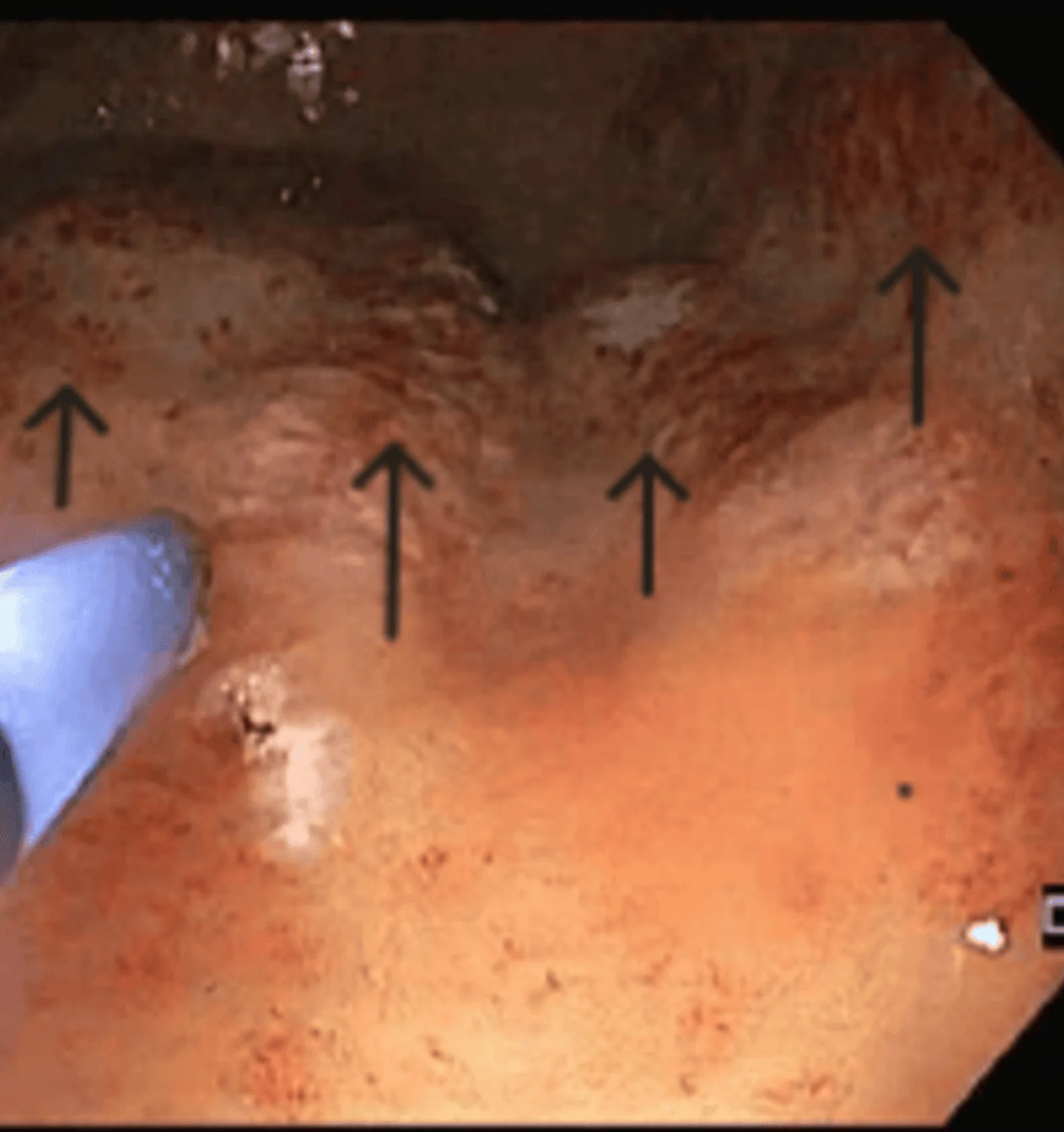
Upper gastrointestinal endoscopy The figure shows multiple diffuse, punctate, erythematous, ectatic vascular lesions (black arrow) involving the antrum, consistent with gastric antral vascular ectasia.

Differential diagnosis

The prolonged history of peripheral neuropathy with recurrent trophic ulceration initially raised the possibility of diabetic peripheral neuropathy. However, the severity of neuropathy was disproportionate to the duration and metabolic control of diabetes and could not adequately explain the progressive localized skin sclerosis, pulmonary involvement, or gastrointestinal manifestations.

Leprosy was considered because of the presence of peripheral neuropathy and trophic ulceration. However, the absence of characteristic skin lesions, negative clinicopathological findings, and the presence of systemic autoimmune manifestations argued strongly against this diagnosis.

Chronic inflammatory demyelinating polyneuropathy was another important consideration because of the chronic progressive neuropathy. Nevertheless, the coexistence of cutaneous sclerosis, pulmonary fibrosis, pulmonary hypertension, positive autoimmune serology, and characteristic histopathology favored an underlying connective tissue disease.

Mixed connective tissue disease was also considered because of strong anti-RNP antibody positivity. However, the predominant clinical phenotype, histopathological evidence of dermal sclerosis, gastrointestinal involvement, cardiopulmonary manifestations, and characteristic cutaneous findings were more consistent with SSc with overlap autoimmune features.

Final diagnosis

Based on the clinical presentation, serological profile, imaging findings, neurophysiological studies, and skin biopsy, a diagnosis of SSc with overlap autoimmune features was established. The disease was complicated by ILD, pulmonary arterial hypertension, moderate pericardial effusion, gastric antral vascular ectasia, chronic mixed sensorimotor peripheral neuropathy with bilateral carpal tunnel syndrome, and Charcot neuroarthropathy of the left foot.

Treatment and follow-up

The patient was managed using a multidisciplinary approach involving rheumatology, pulmonology, gastroenterology, neurology, and rehabilitation medicine. Treatment was directed toward controlling systemic disease activity while addressing individual organ involvement.

For SSc with overlap connective tissue disease features, she was initiated on mycophenolate mofetil (MMF) 500 mg twice daily, hydroxychloroquine 200 mg twice daily, and methotrexate 15 mg once weekly. MMF was used particularly for the associated ILD. Mycophenolate is currently recommended as a first-line immunomodulatory treatment for SSc-associated ILD, with evidence supporting its ability to limit pulmonary disease progression.

The patient's pulmonary arterial hypertension (PAH) was treated with ambrisentan 10 mg once daily, an endothelin-receptor antagonist used in the treatment of SSc-associated PAH. Continued cardiopulmonary surveillance was planned, including clinical assessment of exertional dyspnea and appropriate follow-up of pulmonary function and echocardiographic parameters. Current recommendations emphasize specialist assessment and ongoing monitoring of patients with SSc-associated PAH.

The moderate pericardial effusion was managed conservatively, as there was no clinical indication for immediate invasive drainage. Clinical and echocardiographic follow-up was advised to monitor for progression or the development of hemodynamic compromise.

Her gastrointestinal involvement was managed symptomatically. Gastric antral vascular ectasia (GAVE) was treated with oral iron supplementation, with monitoring of hemoglobin and evidence of ongoing gastrointestinal blood loss. She did not require blood transfusion or endoscopic argon plasma coagulation (APC) during the period of follow-up. Her dysphagia was managed conservatively with nutritional advice and dietary modification, with emphasis on maintaining adequate nutritional intake in view of her anorexia and previous weight loss.

The chronic plantar ulcers associated with sensory loss and Charcot arthropathy were managed with regular local wound care and antibiotics when clinically indicated. MCR (microcellular rubber) footwear was prescribed for pressure off-loading, redistribution of plantar pressure, and protection of the deformed foot. Foot-care education and regular assessment for recurrent ulceration, pressure-related injury, and secondary infection were emphasized.

For symptomatic treatment of her longstanding peripheral neuropathy, gabapentin was prescribed. The neurological manifestations were followed clinically and were not attributed solely to diabetes, particularly because the neuropathic symptoms had begun approximately ten years before the diagnosis of diabetes mellitus. The coexisting bilateral carpal tunnel syndrome was considered an additional component of her peripheral nerve involvement.

Her pre-existing hypothyroidism was managed with continuation of thyroxine 50 μg once daily, with periodic thyroid-function monitoring. Long-term follow-up was planned with serial pulmonary function testing, echocardiography, surveillance for progression of pulmonary hypertension, and regular assessment of neurological function and skin disease activity.

## Discussion

SSc typically presents with Raynaud phenomenon and progressive skin thickening. However, atypical manifestations may precede diagnosis by several years, leading to delayed diagnosis. Such delays are associated with irreversible organ damage, particularly pulmonary fibrosis and pulmonary arterial hypertension, which remain the principal causes of mortality [1-3,5].

The most striking feature of the present case was the prolonged interval between the onset of neurological symptoms and the diagnosis of SSc. Distal sensory neuropathy preceded the appearance of classical systemic manifestations by nearly 20 years. Although the nervous system is not traditionally regarded as a primary target organ in SSc, peripheral nerve involvement has increasingly been recognized. Reported neurological manifestations include carpal tunnel syndrome and other entrapment neuropathies, sensory and sensorimotor polyneuropathies, and less commonly, cranial or proximal nerve involvement. Earlier studies demonstrated both clinically apparent and subclinical peripheral nerve abnormalities, suggesting that neurological involvement may have been underestimated [6]. More recent studies have similarly identified polyneuropathy among patients with established SSc, although the reported frequency varies considerably according to the population studied and the diagnostic methods employed. Importantly, peripheral neuropathy in an individual patient cannot automatically be attributed to SSc, as diabetes mellitus, renal dysfunction, nutritional abnormalities, medications, and other common causes may coexist [6].

The relationship between SSc and peripheral neuropathy is probably multifactorial rather than attributable to a single pathogenic mechanism. SSc-associated microvascular disease may compromise the blood supply of peripheral nerves through involvement of the vasa nervorum, resulting in chronic ischemic or hypoxic nerve injury. Immune-mediated mechanisms have also been proposed, although specific neural autoantibodies have not consistently been demonstrated. In addition, progressive fibrosis and tissue remodeling may contribute to nerve compression, while musculoskeletal and soft-tissue changes can predispose to focal entrapment neuropathies such as carpal tunnel syndrome. Thus, vascular injury, immune-mediated nerve damage, fibrosis/compression, and conventional metabolic or mechanical risk factors may interact in individual patients. A systematic review found peripheral neuropathy to be associated with factors including advanced diffuse disease, calcinosis, ischemia of the vasa nervorum, iron deficiency anemia, and uremia, further supporting a multifactorial model [6].

This emerging understanding is clinically relevant because neuropathy may occasionally be present when the more recognizable manifestations of SSc are subtle or have not yet been identified. In a patient with chronic neuropathy, particularly when accompanied by trophic ulceration, unexplained skin thickening or sclerosis, pigmentary abnormalities, Raynaud-type vascular symptoms, gastrointestinal dysfunction, or evolving cardiopulmonary disease, the possibility of an underlying connective tissue disorder should therefore be reconsidered. At the same time, the temporal relationship between neuropathy and other SSc manifestations may be highly variable, and establishing direct causality remains challenging.

An important strength of this case is the systematic exclusion of more common causes of chronic peripheral neuropathy. Diabetic neuropathy was initially considered because the patient subsequently developed type 2 diabetes mellitus. However, careful chronological assessment demonstrated that neuropathic symptoms had preceded the diagnosis of diabetes by approximately 10 years, making diabetes an unlikely primary cause of her neurological disease. Furthermore, the severity of neuropathy, recurrent trophic ulceration, progressive localized skin sclerosis, pulmonary involvement, and autoimmune serological profile could not be explained solely by diabetic neuropathy.

Hansen’s disease (leprosy) represented another major diagnostic consideration because of the presence of peripheral neuropathy and recurrent trophic ulceration. Nevertheless, the patient lacked characteristic hypoanesthetic skin lesions, and histopathological examination of the skin biopsy demonstrated dense dermal sclerosis without evidence of granulomatous inflammation or features suggestive of Hansen’s disease, effectively excluding this important differential diagnosis. The later evolution of pulmonary arterial hypertension, ILD, gastric antral vascular ectasia, and characteristic autoimmune serology further established SSc as the unifying diagnosis [3,4].

The serological profile in this patient was also noteworthy. Although anti-topoisomerase I (Scl-70) and anticentromere antibodies remain the most characteristic serological markers of SSc, alternative autoantibody profiles are well recognized in overlap syndromes. Strong anti-Ro52 positivity has been associated with ILD, inflammatory myopathy, and more severe systemic disease, whereas anti-RNP antibodies raise the possibility of overlap connective tissue disease. The coexistence of anti-Ro52, anti-RNP/Sm positivity, rheumatoid factor positivity, and mildly elevated serum creatine phosphokinase suggested an overlap autoimmune phenotype despite the absence of clinically overt inflammatory myopathy [4,5].

The cutaneous manifestations in our patient were equally unusual. Rather than diffuse symmetrical skin involvement, sclerosis remained predominantly localized to the left lower limb below the knee, accompanied by edema, induration, hyperpigmentation, recurrent ulceration, and progressive deformity. This asymmetric presentation contributed substantially to the diagnostic delay. However, the characteristic salt-and-pepper pigmentation together with histopathological evidence of dense dermal collagen deposition strongly supported SSc [10].

Hypothyroidism, present in our patient, has been reported in some patients with SSc and may result from thyroid fibrosis or associated autoimmune thyroid disease [11].

Cardiopulmonary involvement remains the principal determinant of long-term prognosis in SSc. Our patient demonstrated both ILD and pulmonary arterial hypertension together with moderate pericardial effusion. High-resolution computed tomography revealed bilateral ground-glass opacities, interlobular septal thickening, traction bronchiectasis, and pulmonary artery dilatation, while pulmonary function testing demonstrated mixed ventilatory impairment. These findings emphasize the need for routine cardiopulmonary screening in all patients with SSc, as early recognition facilitates timely immunosuppressive therapy and targeted pulmonary vasodilator therapy [7,8,12].

Gastrointestinal involvement occurs in up to 90% of patients with SSc, with the esophagus being the most frequently affected organ. Progressive dysphagia in our patient was attributed to scleroderma-related esophageal dysmotility. Gastric antral vascular ectasia (GAVE), identified on upper gastrointestinal endoscopy, represents another characteristic vascular manifestation and is an important cause of chronic occult gastrointestinal blood loss and iron deficiency anemia. Early recognition permits effective endoscopic therapy and reduces transfusion requirements [9].

Another particularly unusual feature of this case was the presence of Charcot neuroarthropathy. Neuropathic arthropathy is classically associated with diabetes mellitus, tabes dorsalis, and syringomyelia, whereas its occurrence in SSc is exceptionally rare. In our patient, longstanding untreated peripheral neuropathy likely resulted in repetitive microtrauma, progressive joint destruction, and severe deformity of the left foot. This combination of chronic neuropathy, unilateral localized sclerosis, and Charcot arthropathy further emphasizes the exceptional nature of this presentation.

This case underscores several important clinical lessons. First, peripheral neuropathy may rarely represent the earliest manifestation of SSc and may precede classical cutaneous features by many years (Table 2). Second, chronic unexplained neuropathy associated with recurrent trophic ulceration or progressive localized skin sclerosis should prompt evaluation for an underlying connective tissue disease after excluding common causes such as diabetes mellitus and Hansen’s disease. Finally, comprehensive multidisciplinary assessment remains essential because delayed diagnosis may permit progressive involvement of the lungs, gastrointestinal tract, heart, and peripheral nervous system before appropriate therapy is initiated.

**Table 2:** Published cases/systematic review of systemic sclerosis presenting with peripheral neuropathy compared with the present case SSc: systemic sclerosis; ILD: interstitial lung disease; GI: gastrointestinal; Scl-70: anti-topoisomerase I antibody; ACA: anticentromere antibody; CTS: carpal tunnel syndrome; PAH: pulmonary arterial hypertension; GAVE: gastric antral vascular ectasia; RF: rheumatoid factor; Anti-Ro52: anti-Ro52 antibody; Anti-RNP/Sm: anti-ribonucleoprotein/Smith antibody.

Study	Age/sex	Neuropathy preceding SSc	Type of neuropathy	Other SSc manifestations	Autoantibodies	Outcome
Ho and Reveille, 2003 [4]	Review	Rare	Sensorimotor	Skin sclerosis, ILD	Variable	Diagnosis after skin disease
Denton et al., 2017 [2]	Review	Uncommon	Mixed neuropathy	Pulmonary, GI disease	Scl-70/ACA	Variable
Allanore et al., 2015 [3]	Review	Rare	Peripheral neuropathy	Diffuse systemic disease	Variable	Chronic disease
Present case	62/F	20 years	Mixed sensorimotor neuropathy + bilateral CTS + Charcot arthropathy	Localized lower-limb sclerosis, ILD, PAH, GAVE, pericardial effusion	Anti-Ro52+, Anti-RNP/Sm+, RF+	Diagnosis delayed by two decades

## Conclusions

This case highlights the need for diagnostic reassessment when chronic peripheral neuropathy is accompanied by unexplained cutaneous or systemic abnormalities. Peripheral neuropathy is not among the most characteristic presenting manifestations of SSc and may therefore lead to an alternative diagnosis being pursued for years. In our patient, the onset of neuropathic symptoms approximately a decade before the diagnosis of diabetes mellitus, together with trophic ulceration, progressive skin sclerosis, and subsequent cardiopulmonary and gastrointestinal involvement, provided important clues to an underlying connective tissue disease. Skin biopsy further supported the diagnosis and did not demonstrate features suggestive of Hansen’s disease.

The neuropathy in SSc is multifactorial, and its presence should prompt evaluation for vascular, immune-mediated, and other mechanisms rather than being attributed to a single cause. The key diagnostic message is that longstanding neuropathy with trophic ulcers, unexplained skin sclerosis, or emerging multisystem manifestations should trigger reconsideration of connective tissue disease, even when neurological symptoms precede the more typical manifestations of SSc. Early recognition of this evolving clinical pattern may facilitate timely identification of systemic involvement and appropriate multidisciplinary management.
